# P-597. Amezosvatein Demonstrates Non-Inferior Immunogenicity and Superior Tolerability Head-to-Head vs Shingrix in a Phase 2 Herpes Zoster Vaccine Trial

**DOI:** 10.1093/ofid/ofae631.795

**Published:** 2025-01-29

**Authors:** Lisa R Shelton, Jon Yankey, Soren Gantt, Guy De La Rosa, David A G Skibinski, George Simeon, David Miller

**Affiliations:** Curevo Vaccine, Bothell, Washington; Curevo Vaccine, Veristat Inc., Bothell, Washington; Université de Montréal, Montreal, Quebec, Canada; Curevo Vaccine, Bothell, Washington; Curevo Vaccine, Bothell, Washington; Curevo Inc., Bothell, Washington; Curevo Vaccine, Bothell, Washington

## Abstract

**Background:**

Low uptake and significant second-dose avoidance for herpes zoster (HZ) vaccination are behaviors linked to poor tolerability of the existing AS01_B_-adjuvanted recombinant gE herpes zoster vaccine, Shingrix, and represent an unmet medical need for a less reactogenic herpes zoster vaccine. Amezosvatein (CRV-101) is an investigational recombinant gE vaccine using a third-generation synthetic adjuvant (SLA-SE) targeting the human TLR4 receptor with improved tolerability and non-inferior immunogenicity.

Humoral Immunogenicity: Anti-gE Antibody VRR and GMFR, and Anti-VZV Neutralizing Antibody GMFR

**Figure:**
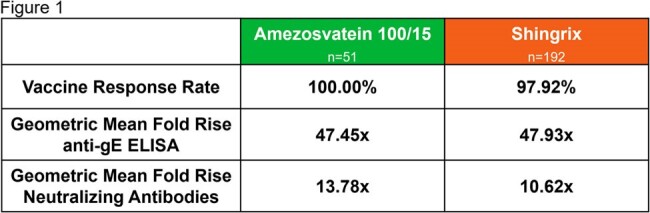

Vaccine Response Rate, defined as a 4-fold rise in antibody concentration from baseline, Geometric Mean Fold-Rise (GMFR) in anti-gE ELISA from baseline, and fold rises in neutralizing antibody were identical or higher for amezosvatein

**Methods:**

A randomized, observer-blind, active-controlled, multi-center Phase 2 trial of amezosvatein versus Shingrix enrolled 876 participants aged 50+. Participants received two injections of either Shingrix or amezosvatein at up to 100 μg of gE antigen with 5, 10, or 15 μg of SLA-SE. The primary endpoints of the study compared safety/tolerability and non-inferiority of immunogenicity of amezosvatein vs Shingrix.

Amezosvatein demonstrates superior tolerability to Shingrix

**Figure:**
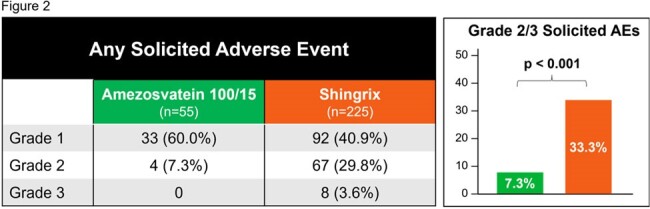

Amezosvatein demonstrated a clinically superior tolerability profile compared to Shingrix as measured by Grade 2/3 solicited local and systemic adverse events.

**Results:**

Amezosvatein at 100 μg gE/15 μg SLA-SE demonstrated immunogenicity non-inferior to Shingrix at Day 84 as measured by anti-gE ELISA Geometric Mean Concentration (50,000 mIU/mL vs. 56,959 mIU/mL, adjusted ratio 0.89, 80% CI 0.79; 1.01). Vaccine Response Rate, defined as a 4-fold rise in antibody concentration from baseline, Geometric Mean Fold-Rise (GMFR) in anti-gE ELISA from baseline, and fold rises in neutralizing antibody were identical or higher for amezosvatein (Figure 1).

Amezosvatein demonstrated a clinically superior tolerability profile compared to Shingrix as measured by Grade 2/3 solicited local and systemic adverse events. Grade 2/3 solicited AEs were reported by 7.3% of amezosvatein recipients versus 33.3% of Shingrix recipients. This difference was statistically significant in a *post hoc* analysis (p< 0.001) (Figure 2).

**Conclusion:**

Our findings suggest amezosvatein has been shown as efficacious with a potentially better tolerability profile, making it a potentially-attractive alternative for the prevention of herpes zoster. Global Phase 3 trials of amezosvatein are anticipated to begin shortly.

**Disclosures:**

**Lisa R. Shelton, ARNP**, Curevo Vaccine: Salary|Curevo Vaccine: Stocks/Bonds (Private Company) **Soren Gantt, MD PHD MPH FRCPC**, Altona Diagnostics: Grant/Research Support|Curevo Vaccine: Advisor/Consultant|GSK: Advisor/Consultant|GSK: Grant/Research Support|Merck: Advisor/Consultant|Merck: Grant/Research Support|Meridian Biosciences: Grant/Research Support|Moderna: Advisor/Consultant|Moderna: Grant/Research Support|Pfizer: Grant/Research Support|VBI: Grant/Research Support **Guy De La Rosa, MD**, Curevo Vaccine: Employee|Curevo Vaccine: Stocks/Bonds (Private Company) **David A. G. Skibinski, PhD**, Curevo Vaccine: Salary|Curevo Vaccine: Stocks/Bonds (Private Company) **George Simeon, MPH**, Curevo Inc.: Board Member|Curevo Inc.: salary|Curevo Inc.: Stocks/Bonds (Private Company) **David Miller, BA**, Curevo Vaccine: Employee|Curevo Vaccine: Stocks/Bonds (Private Company)

